# Large trees are surrounded by more heterospecific neighboring trees in Korean pine broad-leaved natural forests

**DOI:** 10.1038/s41598-018-27140-7

**Published:** 2018-06-14

**Authors:** Hongxiang Wang, Hui Peng, Gangying Hui, Yanbo Hu, Zhonghua Zhao

**Affiliations:** 1 Research Institute of Forestry, Chinese Academy of Forestry, Key Laboratory of Tree Breeding and Cultivation, State Forestry Administration, Beijing, 100091 China; 2 Fengyangshan National Nature Reserve, Longquan, Zhejiang province 323700 China

## Abstract

Negative conspecific density dependence is one of the principal mechanisms affecting plant performance and community spatial patterns. Although many studies identified the prevalence of density dependent effects in various vegetation types by analyzing conspecific spatial dispersal patterns (spatial patterning) of forest trees, interactions between individuals and heterospecific neighboring trees caused by density-dependent effects are often neglected. The effects of negative density dependence lead us to expect that neighbourhood species segregation would increase with increasing tree size and that larger trees would be surrounded by more heterospecific neighbours than would smaller trees. We studied four mapped 1-Ha plots on Changbaishan Mountain in North-eastern China and used marked point pattern analysis to explore whether trees of different sizes exhibited differences in neighbourhood species segregation; we also determined whether larger trees were more likely to have heterospecific neighbours than smaller trees were. Our results show that bigger trees generally have higher species mingling levels. Neighborhood species segregation ranged from lower than expected levels to random or nearly random patterns at small scales as tree size classes increased under heterogeneous Poisson null model tests. This study provides some evidence in support of negative density dependent effects in temperate forests.

## Introduction

In the time since Janzen^1^ and Connell^2^ described the impairing performance of conspecific neighbors in forest communities due to host-specific pathogens, herbivores, seed predators and intraspecific competition, negative conspecific density dependence has become recognised as important in the maintenance of species diversity. Numerous studies were conducted to provide evidence of density dependent effects for the explanation of coexistence of a great number of species in forest ecosystems^3–7^. Two methods are often used to detect negative density dependence in forests. One involves monitoring the growth and mortality of a particular species as well as the development of its seedlings (because trees are more sensitive to competition during early life stages)^8–11^. However, this approach may be insufficient because the reactions of trees to density-dependent effects may not be readily detectable over short time intervals^12^, and seedling mortality can vary with environmental heterogeneity and large scale natural disturbances such as droughts and floods.

Another widely used approach identifies the processes that establish and maintain species biodiversity via detailed analyses of spatial patterns^13^. Differences in spatial structures reflect the complex dynamics involving growth, competition and mortality^14,15^. If density dependence is strong, the pattern created by a focal tree and its neighbours may reflect such effects. Several authors found that density dependence structured and regulated spatial patterns involving trees of different size classes^16–20^. Consistently, conspecific clustering declined with increasing tree size, and the distribution of live trees surviving after density-dependence became more regular^18,20–23^. For example, Zhu *et al*.^20^ compared spatial patterns of different size classes of conspecific trees in a subtropical forest using a case –control approach and found that 83.0% of all evaluated species showed a decline of strength of additional clustering from saplings to juveniles. The statistical methods previously used in efforts to identify negative density-dependent effects often compared the distributional patterns of conspecific species, but did not consider neighbourhood species identities in relation to different tree sizes. After the development of spatial statistical models useful to evaluate forest ecology, the focus has shifted to consider not only the spatial organization of tree positioning but also the effects of tree species and size and the spatial correlations among such attributes. For example, Ledo^24^ and Ledo *et al*.^25^ used an intertype mark correlation function and analyzed pairwise spatial associations between species in relation to tree sizes. Similarly, we sought correlations between individual tree size and neighbourhood species diversity when mingling was in place in a late successional natural forest with the aim of enhancing knowledge about the ecological processes in play.

Strong intra-interaction results in a lower survival of trees that are more crowded; trees within high-density conspecific patches are selectively removed, effectively increasing the distance between conspecifics. Therefore, gaps caused by mortality of conspecific trees may be colonised by new heterospecific neighbours that may enjoy survival advantages when robust intraspecific competition is in play. However, such an effect cannot be detected by comparing the unmarked spatial distribution patterns of the various life stages of conspecific trees. Negative interactions (strong competition for space and resources) can reduce the densities of heterospecific neighbours of large trees^26^. Aggregated species distributions have been recorded in various natural communities, especially at early growth stages^27,28^. If density dependence is in play, intraspecific aggregation emphasises intra- rather than interspecific competition; heterospecific trees may thus enjoy recruitment and survival advantages when growing among cohorts of conspecific trees, and the numbers of other species may increase as conspecific tree size increases. This may be particularly evident around very large trees (e.g. those of diameter at breast height [dbh] >20 cm); such trees significantly affect neighbouring tree recruitment and growth^10,29^. Assuming that density-dependent effects are important in the maintenance of species segregation patterns, we would expect that small trees would exhibit low-level neighbourhood species mingling patterns and that neighbourhood heterogeneity would rise around large focal trees.

Marked point-pattern analysis effectively detects species diversity around a reference tree by using both nearest-neighbor statistics without presenting varying spatial scales or by second-order characteristics with detectable correlation distances of points^30^. In the time since Hui and Gadow^31^ developed several simple and effective nearest-neighbor indices, the mingling index (the proportion of a total of *n* nearest neighbours that are not of the same species as the reference tree) has been widely used to describe forest spatial structure and diversity. An association between individual tree size and the nearest-neighbour mingling level (within a fixed number of neighbourhoods) is directly detected by analysing correlations between the two variables; both can be considered as attributes of forest trees. However, if neighbourhood tree species mingling at various scales is of importance, second-order functions should be applied. Pommerening *et al*.^32^ and Hui and Pommerening^33^ used species mingling as so-called “constructed marks” and developed a mark mingling function useful for comparing spatial species mingling via random assignment of species patterns at specific ecological scales. If both the nearest-neighbour and second-order approaches to neighbourhood species mingling are employed, detailed patterns emerge.

Most density-dependent effects have been described in studies of tropical forests rather than temperate coniferous or broad-leaved mixed forests^3^. We used census data on four 1-Ha plots (map; Materials and Methods) located in an old-growth (almost undisturbed) forest in Northeast China to analyse density dependence as reflected by neighbourhood species segregation around trees of different sizes. We first used the nearest-neighbour approach to explore the relationship between neighbourhood mingling levels and tree size at the community level. Differences in species mingling around small and large trees were directly detected by comparing mingling values and the bivariate distributions of individual tree *dbh* values. In addition, we compared the mark mingling functions of trees of different size classes to explore how species mingling varied by tree size. To determine whether larger trees exhibited greater neighbourhood species mingling than smaller ones, we developed a second-order function to simply calculate mark mingling differences between large and small trees. We apply and explain our new functions using simulated mixed-stand data in which larger trees had more heterospecific neighbours than smaller trees.

In this study, we explored differences in species mingling among trees of different sizes in an old-growth forest in Northeast China. As conspecific neighbourhoods tend to become more diverse with increasing age due to conspecific density dependence, we expected that (1) large trees of Korean pine broad-leaved natural forests would have more heterospecific trees in their local neighbourhoods than would smaller trees and (2) tree neighbourhood species diversity would increase with increasing tree size.

## Results

### DBH-associated mingling bivariate distributions at the community level

**Figure 1:**
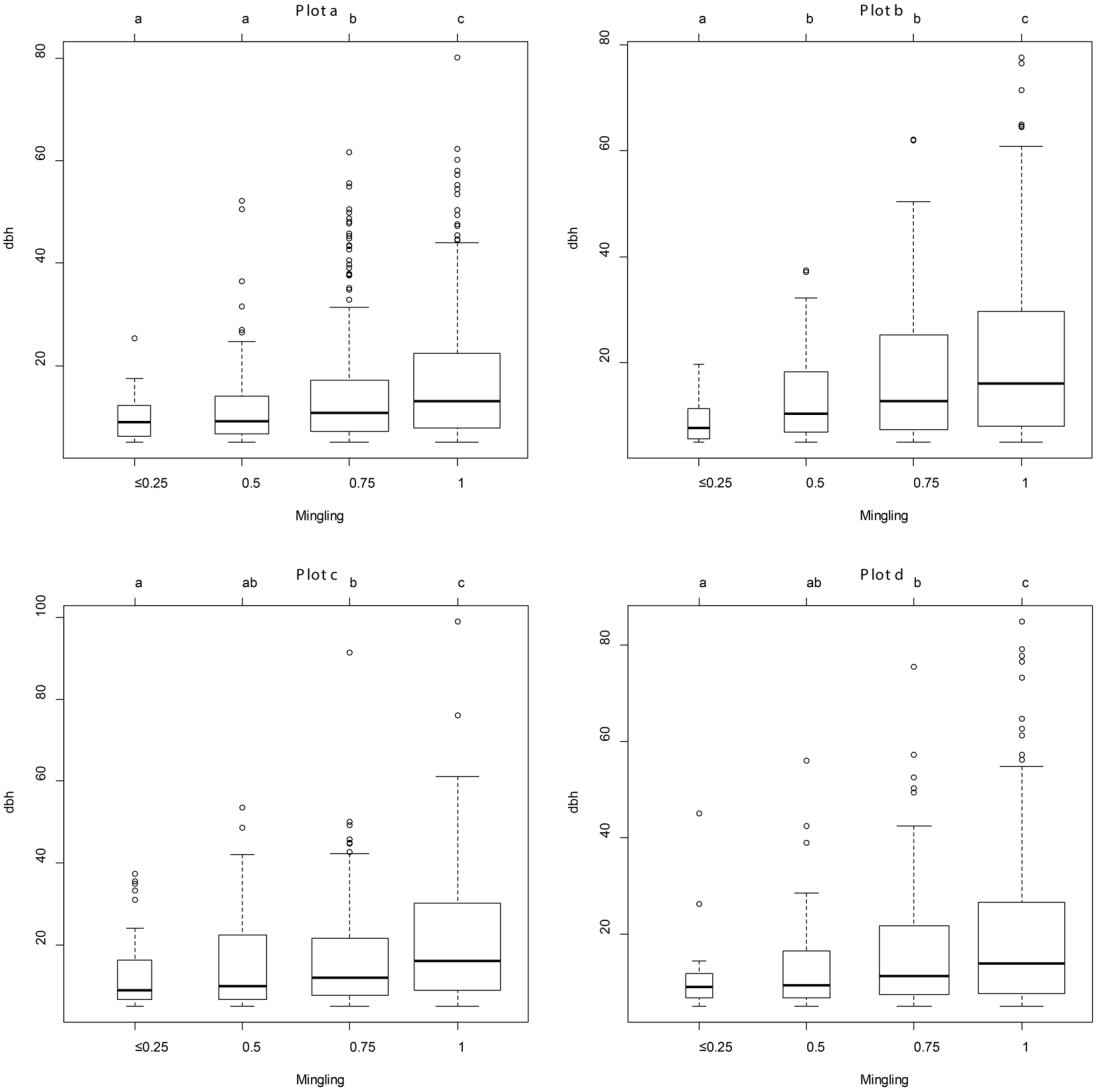
Differences of tree sizes at different mingling levels. Different letters denote significant differences (*p* < 0.05) in *dbh* values when the neighbourhood species mingling levels varied. The four nearest-neighbour trees of an individual tree were used to construct the mingling index. If a reference tree had no or only one heterospecific neighbor, the mingling level was 0 or 0.25. If all four neighbors were heterospecific, the mingling level was 1 (high mingling). Trees of mingling levels 0 and 0.25 were grouped into a single category.

The relationships between mingling level and *dbh* are presented in Fig. 1 for the four plots. The Wilcoxon Rank Sum Test revealed significant differences in the reference tree sizes among the four mingling levels (*p* = 1.15 × 10^−8^, 3.75 × 10^−6^, 2.63 × 10^−6^, and 4.53 × 10^−6^ for the four plots, respectively). Thus, increasing tree size was generally associated with enhanced mingling when the mingling levels were compared in a pairwise manner. In all four plots, trees with four heterospecific neighbors (*M* = 1) were significantly larger than trees exhibiting lower mingling levels.

**Figure 2:**
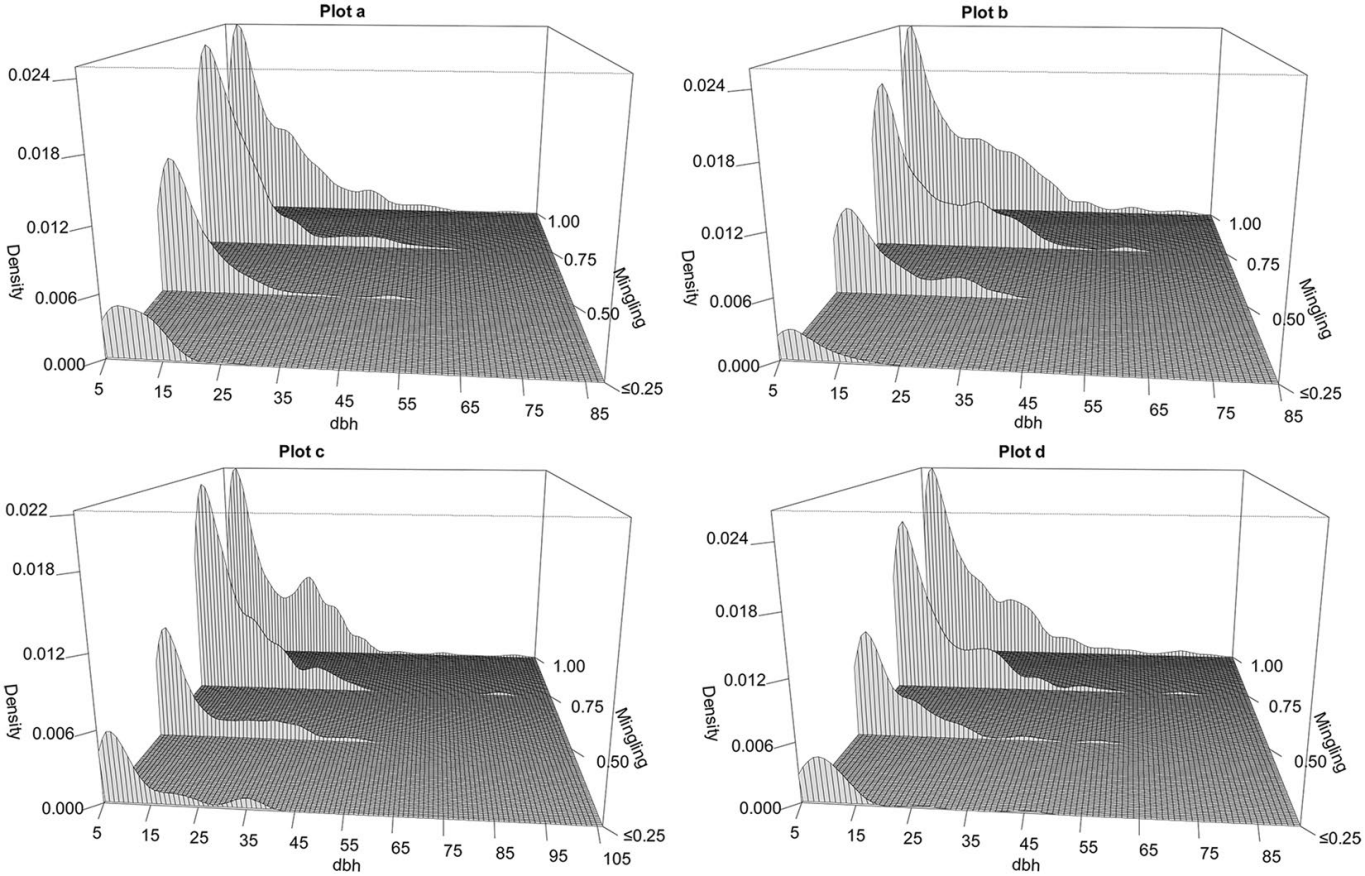
Bivariate distributions of tree sizes and mingling. Tree *dbh* size is a continuous variable, whereas the associated mingling is a discrete variable assuming one of four values: 0 and 0.25, 0.5, 0.75, or 1. The four nearest-neighbour trees were used to construct the mingling index. Trees with mingling levels of 0 and 0.25 were grouped into a single category.

The bivariate distributions of the mingling indices and tree sizes (Fig. 2) also exhibited the trend shown by Fig. 1. Large trees (*dbh* > 25 cm) were almost exclusively associated with mingling levels *M* = 0.75 or *M* = 1.

Both Fig. 1 and Fig. 2 show that large trees (*dbh* > 25 cm) had more heterospecific neighbors while the low mingling levels were mainly associated with small trees.

### Neighbourhood species diversity of trees of different size classes

**Figure 3:**
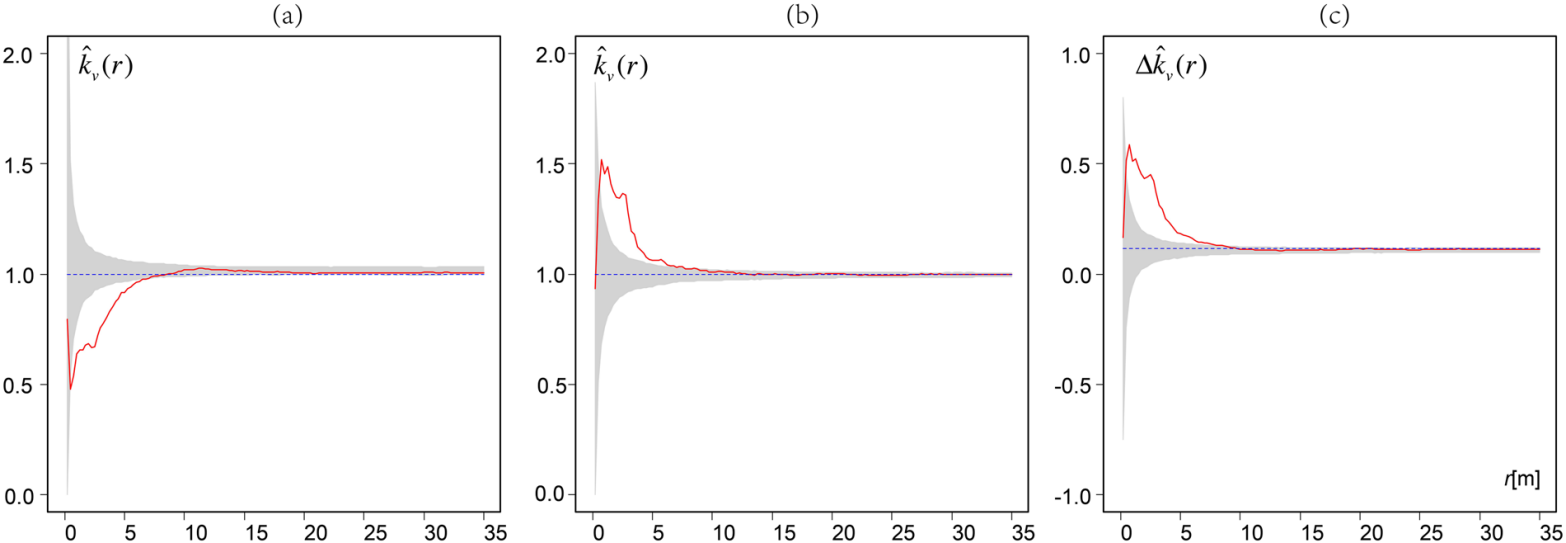
Mark mingling functions (${\hat{k}}_{v}(r)$) of simulated data for small trees (**a**), large trees (**b**), and their difference (${\rm{\Delta }}{\hat{k}}_{v}(r)$) in terms of non-normalised mark mingling functions (**c**). Solid curves: observed mark mingling functions. Envelopes (grey areas): independent marking patterns constructed using the 2.5% and 97.5% quantiles of 1,000 Monte Carlo simulations. Dotted lines: expected mingling values under independent marking patterning. *Note:* It indicates an aggregation of similar tree species if mark mingling function ${\hat{k}}_{v}(r)$ curves fall below the simulation envelops. Different tree species are aggregated if ${\hat{k}}_{v}(r)$ curves are above the simulation envelops. Larger trees exhibit greater neighbourhood mingling than do smaller trees if the ${\rm{\Delta }}{\hat{k}}_{v}(r)$ curves lie above the simulation envelopes.

Mingling mark functions were used to compare neighborhood species mingling among different tree size classes at different scales for both simulated plot and real plots. In the simulated plot, we found that neighbourhood species mingling of small trees (*dbh* ≤ 25 cm) was very aggregated (Fig. 3 left), whereas other tree species were more evenly distributed around large trees (*dbh* > 25 cm) at close range (0–10 m) (Fig. 3 centre). The differences in neighbourhood mingling between the two size classes were greater than expected at 0–8 m (Fig. 3 right).

**Figure 4:**
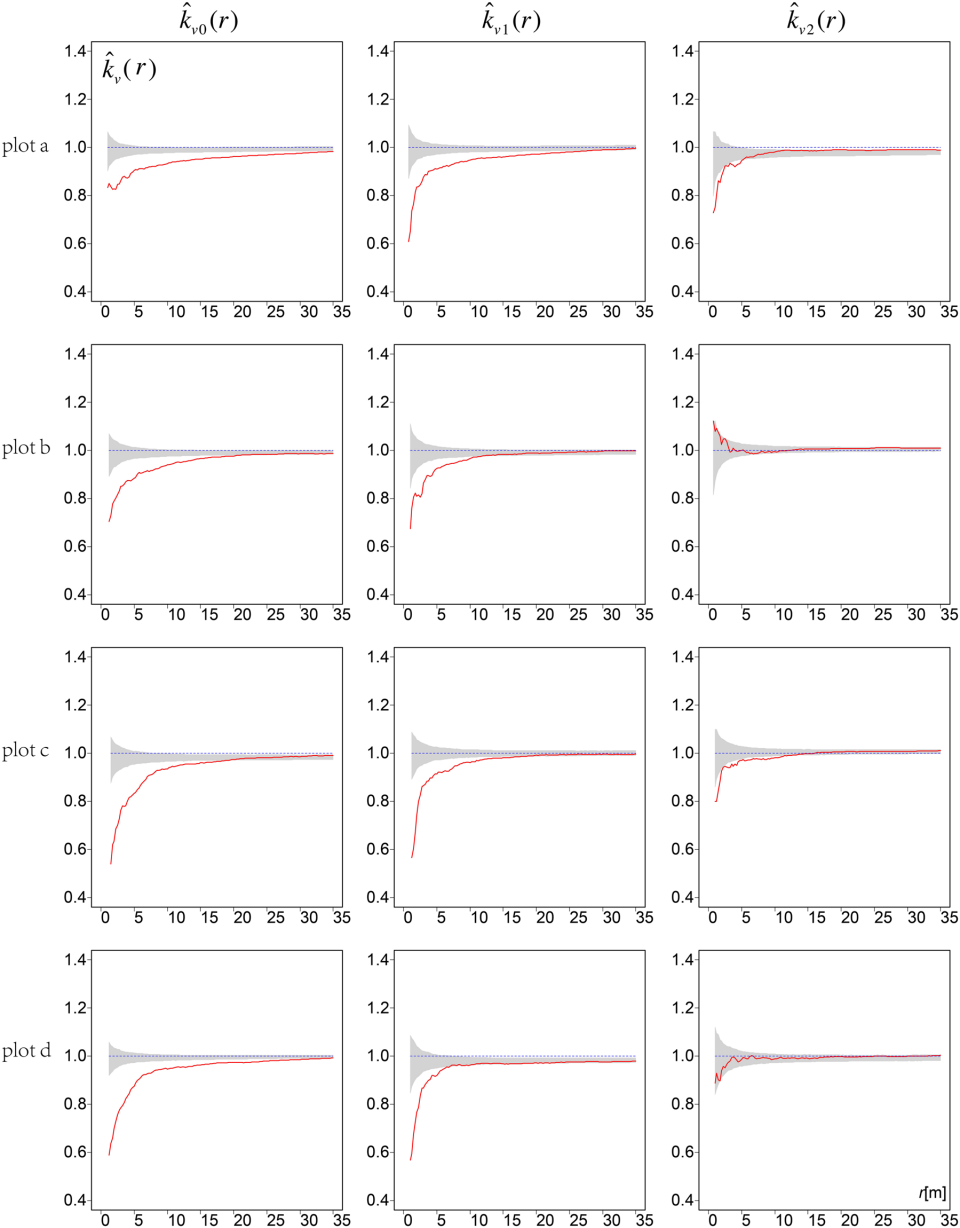
Mark mingling functions (${\hat{k}}_{v}(r)$) for trees of different size classes. Solid curves: observed mark mingling functions. Envelopes (grey areas) were constructed using the 2.5% and 97.5% quantiles of 1,000 Monte Carlo simulations. Heading subscripts 0, 1, and 2 denote three *dbh* size classes (small, intermediate, and large, respectively). *Note:* Curves lying below the simulation envelopes indicate aggregation of similar tree species. Curves lying above the simulation envelopes indicate aggregations of different tree species. The distributions of tree species are random if the curves lie within the simulation envelopes.

For the four real stand plots, the mingling mark functions for trees of different size classes showed that neighbourhood species mingling of small and medium-sized trees was much lower than expected at small scales and that the extent of mingling increased with increasing distance (Fig. 4). Large trees exhibited random species mingling (plots b and d) or mingling that was close to random in nature (plots a and c).

**Figure 5:**
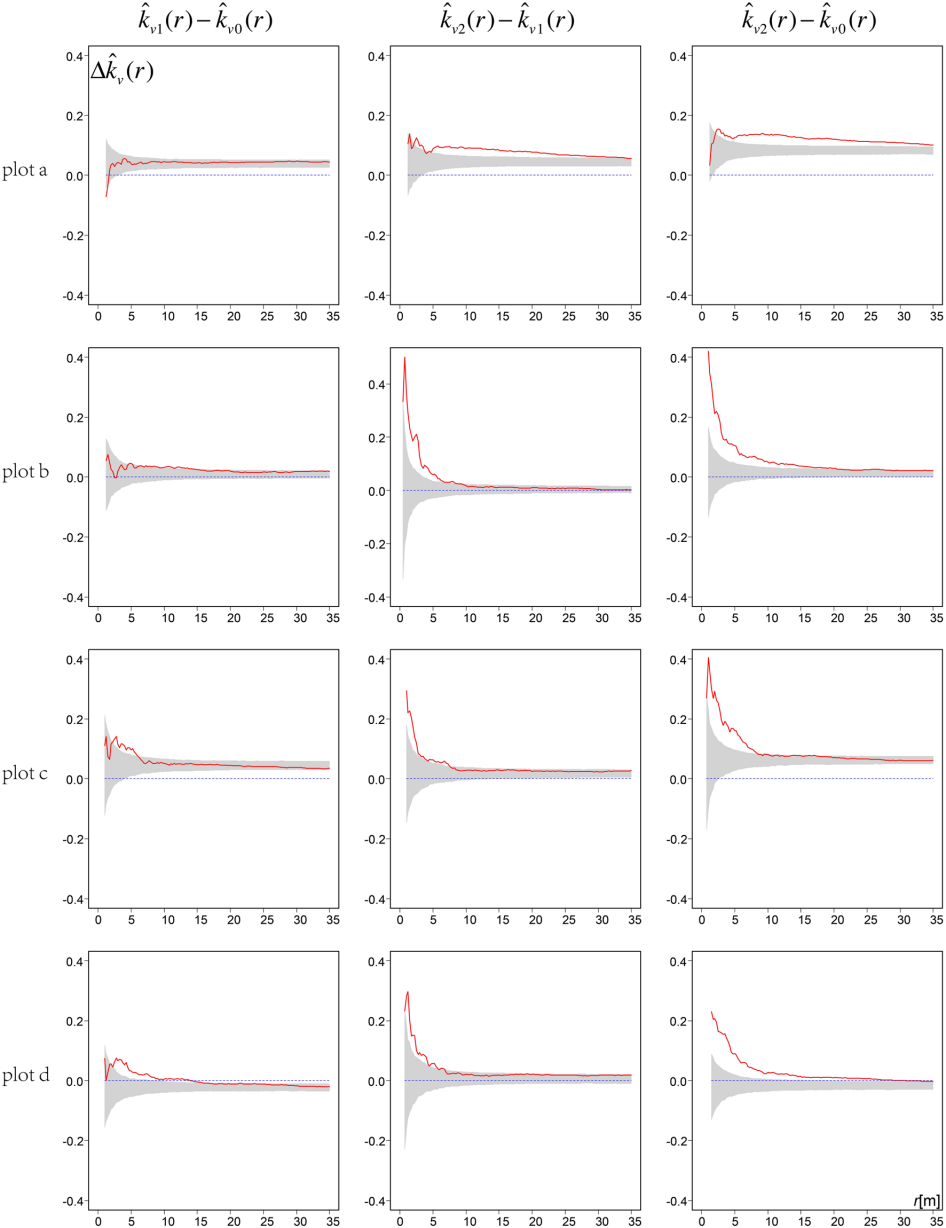
Differences in neighborhood species mingling (${\rm{\Delta }}{\hat{k}}_{v}(r)$) among small, medium and large trees in each plot. Solid curves: observed ${\rm{\Delta }}{\hat{k}}_{v}(r)$; envelopes (grey areas) were constructed from the 2.5% and 97.5% quantiles of 1000 Monte Carlo simulations. Heading subscripts 0, 1, and 2 denote three *dbh* size classes (small, medium, and large, respectively). *Note:* Larger trees exhibit higher neighbourhood mingling than do smaller trees if the curves lie above the simulation envelopes. Curves that lie within the simulation envelopes indicate no differences in neighbourhood mingling between larger and smaller trees.

Generally, neighborhood species mingling increased with increasing tree size (Fig. 4). The differences in mingling levels among the size classes showed that both the large to intermediate (${\hat{k}}_{v2}(r)-{\hat{k}}_{v1}(r)$) and large to small (${\hat{k}}_{v2}(r)-{\hat{k}}_{v0}(r)$) size classes exhibited more mingling than expected by comparison with the simulation results (Fig. 5). The differences between medium and small size classes (${\hat{k}}_{v1}(r)-{\hat{k}}_{v0}(r)$) were not as obvious as those between the large and small (${\hat{k}}_{v2}(r)-{\hat{k}}_{v0}(r)$) size classes in terms of either the extent of deviation or the range of interactions.

The expected differences in neighborhood mingling (${\rm{\Delta }}{\hat{k}}_{v}(r)$) derived from the null model simulations were greater than zero in plots a and c and in the simulated plot, but were approximately zero in plots b and d, attributable to differences in the *dbh* size distributions of abundant and less abundant tree species. Supplementary Figs S1–S4 show the *dbh* size distributions of the species represented by >50 individual trees in any of the four plots. The abundant species (the most common three species) were mainly small (most of *dbh* <20 cm), whereas the less abundant species in plots a and c were generally larger. This was also the case in the simulated plot, where larger trees were less abundant than smaller trees. However, these differences were not obvious in plots b and d.

## Discussion

Many attempts have been made to understand processes and mechanisms that determine tree spatial patterns and the maintenance of diversity in plant communities^7,34^. Negative conspecific density-dependent effects are important in terms of structuring and regulating spatial patterns, as previously revealed by many studies using detailed analyses of tree spatial patterns. However, these point pattern studies only analyzed the conspecific spatial distribution patterns and simply considered tree locations of particular species but neglected the information of interactions among trees of diverse neighborhood species (non-conspecific trees)^16^. If negative intraspecific interactions are strong, these will structure the spatial distribution patterns of conspecific trees and also affect segregation patterns. We addressed this research gap by examining density-dependent effects while considering both intra- and interspecific neighbourhood relationships. We used the species mingling index and mark mingling functions to detect correlations between individual tree sizes and neighbourhood species diversity. Previous studies also employed various intertype second-order functions (such as the intertype mark correlation function) to detect correlations between two types of points (e.g. two species) or to assess the correlation between the sizes of two species^24,25,35,36^. However, such models deal principally with pairwise spatial associations between species; it is better to use a species mingling index or a mark mingling function when analysing spatial segregation patterns in communities containing diverse species.

We explored whether trees at different growth stages were equally surrounded by conspecific or heterospecific neighbours. If the proportions of heterospecific neighbouring trees increase with individual tree size in long-term undisturbed forests, strong intraspecific interactions (i.e. negative conspecific density dependence) are clearly in play. We found that aggregation of identical neighbourhood species on the small scale decreased with increasing tree size, as larger trees were surrounded by more heterospecific neighbours than were smaller trees.

### Nearest-neighbour analysis and second-order characteristics

Both nearest-neighbour mingling analysis (with a fixed number of neighbourhoods) and evaluation of second-order characteristics showed that large trees tended to have more heterospecific neighbours; the mark mingling second-order functions provided detailed information on the spatial scales (mark correlation ranges) and the extent of deviation from independent-marking patterns^33^. Nevertheless, the traditional, simple mingling distribution method readily associated individual tree mingling level with tree size, as shown by both the boxplots and the bivariate distributions (Fig. 1 and Fig. 2). Thus, we suggest that both nearest-neighbour analysis and mark mingling second-order functions reveal interesting and detailed ecological patterns^32,37^. We compared the mark mingling functions of trees of different sizes by roughly dividing all trees into three size groups, as in previous studies^16,38^. However, trunk diameter is a continuous variable, and it is always difficult to define tree size cut-off points objectively. Future studies should develop bivariate mark functions that simultaneously consider both marks (e.g. tree species and size).

### Tree species abundance and size distributions

We examined four 100 × 100-m plots in temperate broad-leaved natural forests at the whole-community level; in all four, neighbourhood species aggregation decreased with increasing tree size class. However, more marked neighbourhood mingling of large than of small trees at the whole-community level may not necessarily indicate dependent mark mingling patterns. This is because of the confounding effects of the different *dbh* size distributions of species that are and are not abundant. If many large trees are less abundant, a large difference in neighbourhood species aggregation between small and large trees may also occur, although tree species marks are in fact randomly distributed. This can be tested using independent marked point process null models that calculate the expected neighbourhood mingling differences between trees of different size classes. In our simulated plot and in real plots a and c, the differences in mark mingling values were clearly greater than zero (Figs 3c and 5), indicating that many large trees were of uncommon species. This was further illustrated when the species *dbh* distributions in each plot were examined (Supplementary Figs S1–S4). We found that large trees (*dbh* > 20 cm) were more likely to be associated with tree species that were not among the three most abundant species in plots a and c *(Tilia tuan Szyszyl*., *Abies holopylla* and *Pinus koraiensis Sieb*.*et Zucc*. in plot a, and *Acer mandshuricum Maxim*., *Carpinus cordata var*. *Chinensis* and *Acer mono Maxim*. in plot c), contributing to mingling levels greater than expected (greater than zero). Such confounding effects can be avoided by distinguishing each tree species. As the populations of most species were too low to allow for species-by-species statistical analysis, we limited our evaluation to the whole-community level. However, analysis using all trees in each plot also indicated that mark-dependent point patterns were evident at the whole-community level.

### Aggregation of similar species of small trees

Many studies have shown that trees of the same species tend to be aggregated in natural communities^27,39^; this has generally been attributed to both dispersal limitations and environmental filtering^28,40^. Such aggregations of small trees at local scales reflect principally dispersal limitations; seeds that cannot readily disperse germinate around their parents^28,41^. Intraspecific aggregation increases the significance of intraspecific, compared to interspecific, competition; therefore, the recruitment and survival of heterospecific trees may be enhanced if they grow among cohorts of conspecific trees. For large trees that have survived for a long time, neighbourhood species segregation was higher or more random than that of small trees, as shown by the relationships between individual mingling levels and tree size (Fig. 1 and Fig. 2). Trees associated with high proportions of neighbouring conspecific trees were mainly of small size. The null model, which removes the heterogeneity, showed that the mark mingling functions shifted from aggregation of similar species to a less aggregated or random pattern as tree size class increased (Fig. 4). These results are consistent with previous work on the spatial patterns of different size classes of conspecific trees, which showed that small trees were more aggregated than large trees^16–18,20^. Thus, large trees may encounter more nearby heterospecific trees.

The differences in mark mingling functions among the *dbh* size classes (Fig. 5) showed that large trees exhibited much higher neighbourhood species segregation than did intermediate-sized and small trees. This means that large class trees (*dbh* > 25 cm) have greater influence on their neighborhood than intermediates or small trees. The great intraspecific influence may come from more encounter probability with its species-specific pests and pathogens^1,2^, and from intense asymmetric competition for scarce nutrient resources available to small conspecific neighbours^5,42^. Such strong effects reduce the probability that seedlings will grow into adults, thus limiting the growth of conspecifics. The results suggest that density-dependent effects potentially regulate neighbourhood species segregation at the whole-community level also.

As the plot areas were small, we focused on the whole community, neglecting differences in marked spatial patterns among certain focal species. In the future, we will examine the neighbourhood species segregation patterns among tree size classes at various vegetation types to explore whether differences among focal species can be reflected by species abundance or functional traits (e.g. growth form, shade tolerance, and/or dispersal mode).

## Materials and Methods

### Study Site and Tree Data

**Figure 6:**
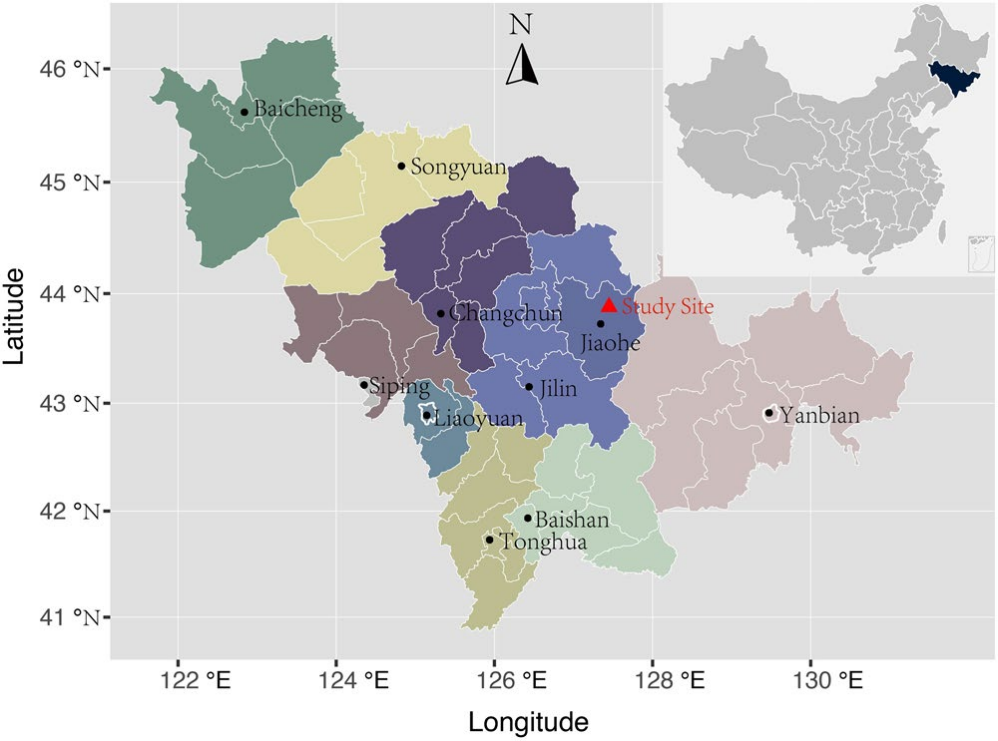
The study sites. The four plots are located in Dongdapo Nature Reserve, Jiaohe, Jilin Province, North-eastern China. The map was produced using the ggplot2^54^ and maptools^55^ packages in R software.

The study site is located in the Dongdapo Nature Reserve (43°51′–44°05′N, 127°35′–127°51′E), which is part of the Zhangguangcai Mountain range extending from north of the Songhua River to south of the Changbaishan Mountains in North-eastern China (Fig. 6). The climate in this region is characterized by dry, windy spring seasons and warm, wet summers, a continental mountain climate affected by monsoon. The annual mean precipitation is 700–800 mm and the distribution of the precipitation during the year is relatively uneven. A relatively wet season extends from June to August, and a dry season from September to May. The annual average temperature is 3.5 °C, and the mean minimum mid-winter temperature −22.2 °C. The soil type is a brown forest soil with abundant accumulation of slightly acidic or neutral humus. The topography is flat or slightly undulating. The forest consists of more than 20 tree species and is dominated by several conifers, i.e. *Pinus koraiensis*, *Abies holophylla*, *Abies nephrolepis*, and *Picea jezoensis var*. *microsperma*.

This study is based on observations of four 1-Ha field plots in the Dongdapo Nature Reserve, which used to be part of the experimental forest farm of Jilin Forestry College. Forest management ceased 50 years ago. All live trees with a *dbh* exceeding 5 cm were tagged, mapped, measured and their species identified.

### Species mingling

We define mingling as the proportion of *n* nearest neighbors that belong to a different species than the species of a particular reference tree^14,43^. Mingling expresses the extent of spatial mixing or segregation, as follows:

1 $${M}_{i}={\bf{1}}({m}_{1}\ne {m}_{2})$$

where *m*_1_ and *m*_2_ are the marks at the tree locations within a radius *r* of a reference tree. **1**(.) is an indicator function that returns the value of 1 if the condition in the brackets is fulfilled, i.e., if the species of a neighboring tree is different from that of a reference tree, and 0 otherwise^33^. This function is also used as the test function for constructing the mark mingling function in the following section.

*M*_*i*_ can be applied to analyse different numbers, *n*, of neighboring trees (e.g. three or four neighbours)^44^. The greater the value of *M*_*i*_, the greater the local species diversity. Here, we used the four nearest-neighbour trees to calculate mingling values, as these neighbours interacted most intimately with the reference tree. This number has been widely used to describe the structures of natural forests^37,45,46^. Thus, for each reference tree, *M*_*i*_ may assume one of five possible values (0, 0.25, 0.5, 0.75, or 1), allowing analysis of the response of reference trees to different neighbourhoods. We evaluate the relationship of different mingling levels with reference tree sizes. As reference trees with no heterospecific neighbors (i.e. *M*_*i*_ = 0) numbered <5% of the total, we grouped trees with mingling levels of 0 and 0.25 into a single category. Statistical analysis was performed using the Wilcoxon Rank Sum Test in the *npar* package of R statistical software^47^.

A bivariate distribution is presented to visualize the relationship between the continuous variable “reference tree size” and the associated discrete variable “mingling”. We used the Gaussian kernel to estimate the probability density functions. The diagrams were generated using the *persp* function of R software.

### Mark mingling function

Neighborhood mingling of particular reference trees was also investigated at varying scales using cumulative mark-correlation functions. These functions can be derived from the corresponding test function *t* as a natural generalization of Ripley’s *K*-function. To our knowledge, Pommerening *et al*.^32^ and Hui and Pommerening^33^ were the first to develop a mark mingling function based on the general concept of a mark correlation function. As our original aim was to find the proportions of heterospecific neighboring trees around individual reference trees of different size within a circle of varying radius *r*, it was necessary to present the cumulative mark-correlation function in analogy to Ripley’s *K*-function which is consistent with our hypothesis. A cumulative function counts all the points within a certain circle, thus reducing the effects of stochastic noise. Based on Ripley’s^48^ *K*-function and Hui/Pommerening’s^33^ mark mingling function, we derived a cumulative mark mingling function to calculate the second-order characteristics of different tree size classes. A core element of this approach is the test function, which was given as an indicator function ${\bf{1}}({m}_{i}\ne {m}_{j})$ in formula 1.

Based on the test function, an estimator of the normalized cumulative product function ${\hat{K}}_{m}(r)\,$can be defined by analogy to Ripley’s *K*-functions^49^ (see pp. 219–220), as follows:

2 $${\hat{K}}_{m}(r)=\frac{1}{{\hat{l}}^{2}}\frac{1}{A}\sum _{i=1}^{n}\sum _{j=1}^{n,\ne }{\bf{1}}({m}_{i}\ne {m}_{j})\times {\bf{1}}(\parallel {x}_{i}-{x}_{j}\parallel ,r)\times {\omega }_{i,j}$$

where $\hat{l}$ is an estimator of the intensity of points within a subwindow *W*, and A is the area of the observation subwindow. *x*_*i*_ and *x*_*j*_ are two arbitrary points of the point pattern within the observation window. **1** $({m}_{i}\ne {m}_{j})\,\,$ yields a value of 1 if these two points are different species and 0 otherwise. $\parallel {x}_{i}-{x}_{j}\parallel$ is the distance between *x*_*i*_ and *x*_*j*_ and *r* is the focusing scale. **1** $(\parallel {x}_{i}-{x}_{j}\parallel ,r)$ yields a value of 1 if $\parallel {x}_{i}-{x}_{j}\parallel \le r$ and 0 otherwise. ${\omega }_{i,j}$ accounts for translation edge correction^50^, *n* is the number of points of a given pattern.

As the mark-product function ${\hat{K}}_{m}(r)\,$contains information on both the structure of unmarked pattern and the correlation structure of marks^49^, the (non-normalized) cumulative mark mingling function ${\hat{k}}_{m}(r)$ can be obtained by removing the conditional spatial structure of the points (unmarked pattern). Thus, we define a simple interpretation of the cumulative mark mingling function ${\hat{k}}_{m}(r)$ as the proportion of *n* nearest neighbors that are of different species than the reference tree *i* and are located within distance *r* centered at point *i*:

3 $${\hat{k}}_{m}(r)=\frac{{\sum }_{i=1}^{n}{\sum }_{j=1}^{n,\ne }{\bf{1}}({m}_{i}\ne {m}_{j})\times {\bf{1}}(\parallel {x}_{i}-{x}_{j}\parallel ,r)\times {\omega }_{i,j}}{{\sum }_{i=1}^{n}{\sum }_{j=1}^{n,\ne }{\bf{1}}(\parallel {x}_{i}-{x}_{j}\parallel ,r)\times {\omega }_{i,j}}$$

The normalized cumulative mark mingling function for different tree size classes therefore is:

4 $${\hat{k}}_{m}(r)=\frac{1}{E{M}_{d}}\frac{{\sum }_{i=1}^{n}{\sum }_{j=1}^{n,\ne }{\bf{1}}({m}_{i}\ne {m}_{j})\times {\bf{1}}(\parallel {x}_{i}-{x}_{j}\parallel ,r)\times {\omega }_{i,j}}{{\sum }_{i=1}^{n}{\sum }_{j=1}^{n,\ne }{\bf{1}}(\parallel {x}_{i}-{x}_{j}\parallel ,r)\times {\omega }_{i,j}}$$

*i* is the point associated with a certain tree *dbh* size class *d* (i.e. trees of small, intermediate or large size classes) while *j* is an arbitrary point of the point pattern in the observation pattern. *EM*_*d*_ is the expected neighborhood species mingling for a certain size class, obtained as the sum of weighted mingling values of each species within a certain size class. We first calculated the expected mingling values for each species and then multiplied these by the proportion of each species in a certain size class. Thus, *EM*_*d*_ is estimated as

5 $$E{M}_{d}=\sum _{i=1}^{s}\frac{{n}_{di}(n-{n}_{i})}{{n}_{d}(n-1)}$$

where s, the number of species, *n*, the number of trees in the observation window, *n*_*i*_, the number of trees of species *i*, and *n*_*d*_, the number of trees of a certain size class in the observation window, *n*_*di*_, the number of trees of species *i* in a *dbh* size class. Therefore, $\frac{(n-{n}_{i})}{(n-1)}$ estimates expected mingling of a species in the whole observation window^51^, and $\frac{{n}_{di}}{{n}_{d}}$ is the estimator of the proportion of species *i* belonging to a certain size class.

We calculate the cumulative mark mingling functions for trees of small, intermediate and large size. All trees were classified as described previously^16,38^: large trees (*dbh* > 25 cm), medium (10 cm ≥ *dbh* ≥ 25 cm), and small (5 cm ≥ *dbh* > 10 cm). By comparing the cumulative mark mingling functions of each size class with their Monte Carlo simulation envelopes, we detected the extent of neighbourhood mingling within each *dbh* size class. Differences in non-normalised mark mingling functions can be used to evaluate differences in neighbourhood mingling levels between larger [${\hat{k}}_{v2}(r)$; pattern 2] and smaller [${\hat{k}}_{v1}(r)$; pattern 1] size classes. If larger trees have more heterospecific trees in their neighbourhoods, the non-normalised mark mingling functions of larger trees should be greater than those of smaller trees.

The difference in neighbourhood mingling between larger and smaller trees of any particular species, ${\hat{k}}_{v2}(r)-{\hat{k}}_{v1}(r)$, would be expected to be 0 if tree size does not affect such mingling. However, the situation is more complicated when considering the ${\hat{k}}_{v2}(r)-{\hat{k}}_{v1}(r)$ calculations for all trees of different species in observational plots. Larger trees would be expected to exhibit higher neighbourhood mingling than smaller trees, even if the species are randomly distributed, if some relatively less-abundant species feature a large proportion of big trees and more abundant tree species are principally small trees. Trees of less abundant species are more likely to be associated with larger trees, which thus exhibit greater neighbourhood mingling. Hence, ${\hat{k}}_{v2}(r)-{\hat{k}}_{v1}(r)$ should be compared with the Monte Carlo simulation envelope to explore whether dependent marked point patterning is in play. At the whole-community level, large reference trees have more heterospecific neighbours than do smaller trees if the ${\hat{k}}_{v2}(r)-{\hat{k}}_{v1}(r)$ curve lies above the simulation envelope.

### Simulation of dependent marked point patterns

We simulated dependent, spatial marked point patterns in which small trees of the same species were aggregated whereas larger trees of the same species were not (i.e. interspecific tree aggregation). We simulated a homogeneous Poisson processes of intensity λ = 0.1 points/m^2^ within an area of 100 × 100 m; this reflected the actual tree density in the observational plots. Two tree species marks, 1 and 2, were defined; species 1 referred to common small trees (average *dbh* 20 cm) and species 2 to less abundant large trees (average *dbh* 30 cm). Tree dbh values were randomly generated and followed a normal distribution.

Dependent marking, which leads to correlations among tree locations, species and diameter attributes, was employed as described by Pommerening *et al*.^32^. First, if the distance from a tree to the second-nearest neighbour was <*r*_0_ = 2.5 m, the species mark was assigned the value 1, and 2 otherwise. This aggregates similar species. *r*_0_ was set to 2.5 m to generate more trees of species 1 and fewer of species 2. Next, for each tree of *dbh* >25 cm, the nearest neighbour was assigned a species mark different from that of the reference tree. This created high-level neighbourhood mingling around large trees over short distances, but similar species remained aggregated around small reference trees (*dbh* ≤25 cm).

We applied cumulative mark mingling functions to explore the dependence of patterns revealed by the simulated data. Comparison of the observed and the independent mark patterns rendered it possible to ascertain the extent by which dependent mark patterns deviated from independence (a random process). We used a homogeneous Poisson function as the null model based on 1,000 Monte Carlo simulations.

### Null model

When the mark mingling functions assessing neighborhood species segregation were developed, the selection of an appropriate null model was important in terms of point-pattern analysis^49^. The null model used to test existing data detects whether an observed pattern differs significantly from that expected if mark independence (implying random assignment of species/diameter marks) is actually the case.

Neighbourhood mingling quantifies both intra- and interspecific tree–tree interactions. Thus, both intra-specific aggregation and inter-specific segregation trigger low-level neighbourhood mingling, and vice versa. Therefore, use of a null model would be expected to conceal not only interspecific tree–tree interactions but all intraspecific relationships. The homogenous Poisson approach can be used to randomise tree locations, thus eliminating all tree–tree interactions and relationships in homogenous environmental habitats. However, factors influencing intra- and interspecies segregation may extend beyond tree–tree interactions^52^. Species habitat associations and/or habitat heterogeneity (topographic or edaphic) may trigger species aggregation or repulsion, but only at larger scales. To eliminate any influence of habitat heterogeneity on tree spatial distribution, we used Monte Carlo simulations of a heterogeneous Poisson null model in which individuals of target species were distributed by reference to their intensities.

The relationship between the various species and *dbh* size of each tree were retained in the simulations. Only the original locations of the species were redistributed according to the intensity function *λ*(*x*, *y*), which varied by location (*x*, *y*), but the occurrence of any point remained independent of that of any other. The intensity function *λ*(*x*, *y*) was estimated by using a moving window with radius *r* that maintained the large-scale spatial structure but modified the spatial pattern on small scales <*r*. Second-order characteristics of tree to tree interactions are often evident at local scales, usually <20 to 30 meters in forests^52^. We defined *r* to be 20 m by reference to the total area of each study plot; we expected significant departures from the observed patterns reflecting marking independence only at scales <20 m.

All analyses were performed using the R statistical software package. The calculations of mingling indices and mark mingling functions were developed based on the R scripts and C++ files of Pommerening Forest Biometrics Laboratory^53^.

## Electronic supplementary material

Supporting information
