# Supplementary material for: Large trees are surrounded by more heterospecific neighboring trees in Korean pine broad-leaved natural forests
Source: Sci Rep. 2018 Jun 14;8:9149. doi: 10.1038/s41598-018-27140-7 (PMC6002480; doi:10.1038/s41598-018-27140-7)
Supplement: Supplementary file 1 — Supporting information [file 41598_2018_27140_MOESM1_ESM.pdf]

Large trees are surrounded by more heterospecific neighboring trees in Korean pine broad-leaved natural forests

Supporting information

Hongxiang Wang, Hui Peng, Gangying Hui, Yanbo Hu, Zhonghua Zhao

SI Text

Supplementary Figs. S1-S4 presented tree *dbh* size distributions of the species represented by more than 50 individuals in the four plots, respectively. For plot *a*, the most common three speceis (*Carpinus cordata var. chinensis*, *Acer mono Maxim.* and *Ulmus pumila L.*) exhibited greater proportion of small trees than the relatively less abundant species (*Tilia tuan Szyszyl.*, *Abies holopylla* and *Pinus koraiensis Sieb.et Zucc.*), i.e., big trees (*dbh*>20 cm) contained larger proportion of less abundant species than small trees. For plot *c*, it also showed that relatively less abundannt speceis (*Ulmus pumila L.* and *Juglans mandshurica Maxim*) exhibited larger proportion of large trees (*dbh* > 20 cm) than abundant species (*Acer mandshuricum Maxim.*, *Carpinus cordata var. Chinensis* and *Acer mono Maxim.*). However, there was no clear evidence showing big trees (*dbh* > 20 cm) were likely associated with less abundant tree species in plot *b* and *d*.

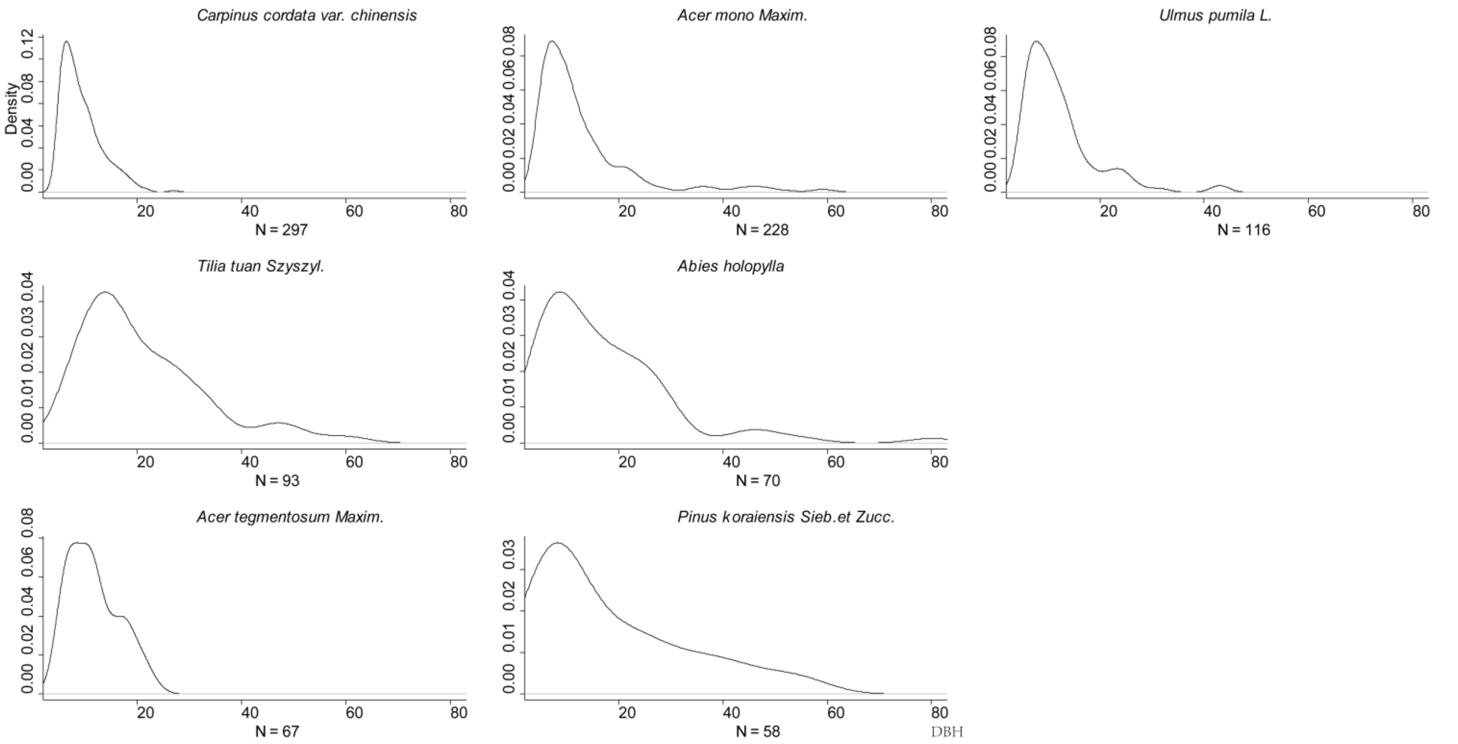

Supplementary Fig. S1 Tree dbh size distributions for species represented by more than 50 individuals in plot a.

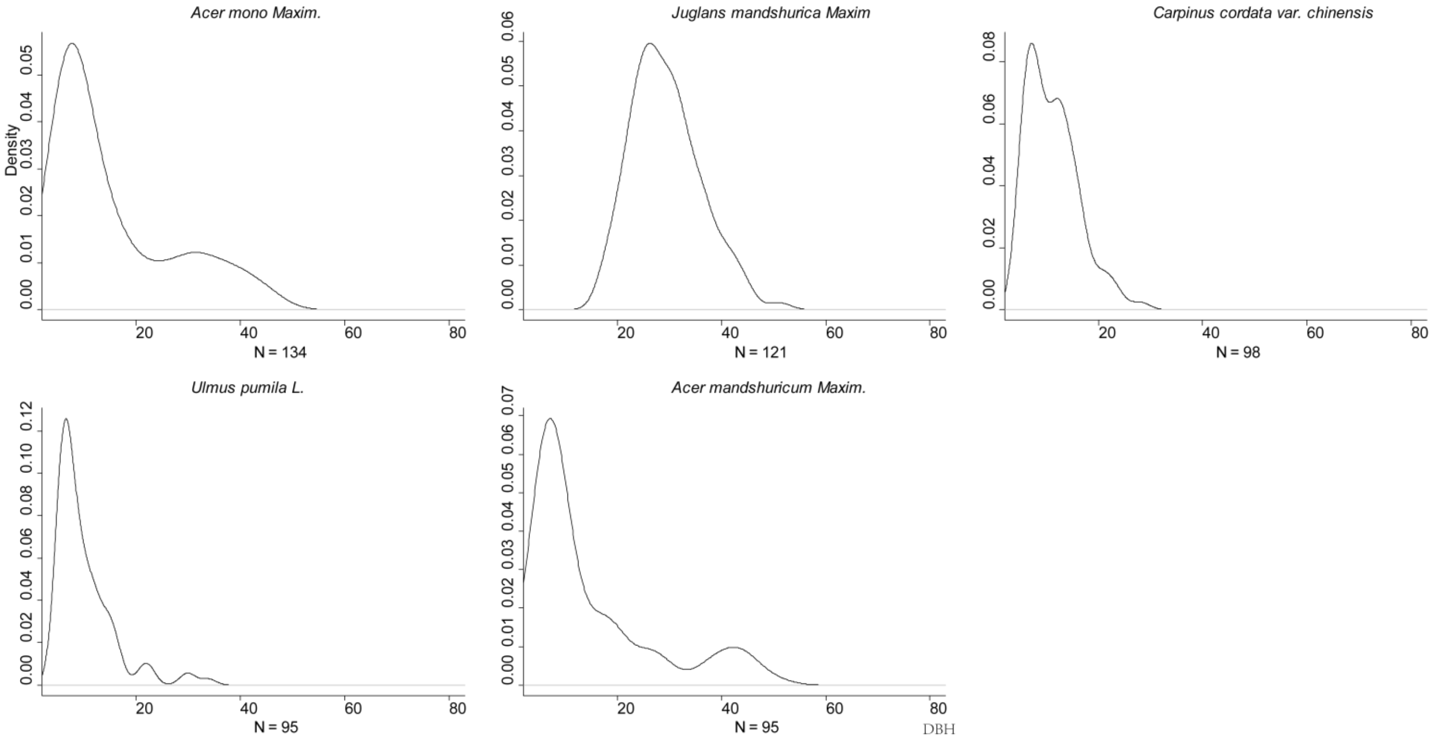

Supplementary Fig. S2 Tree dbh size distributions for species represented by more than 50 individuals in plot b.

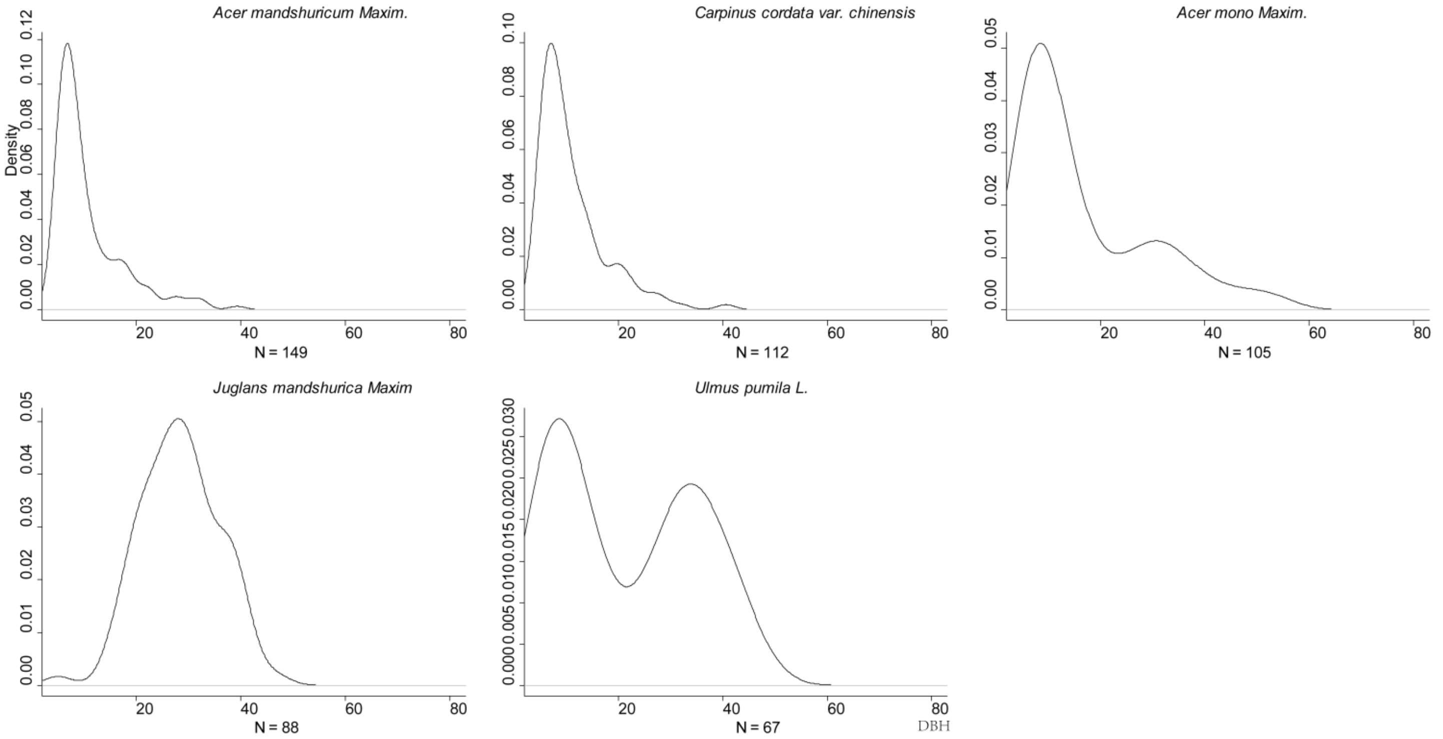

Supplementary Fig. S3 Tree dbh size distributions for species represented by more than 50 individuals in plot c.

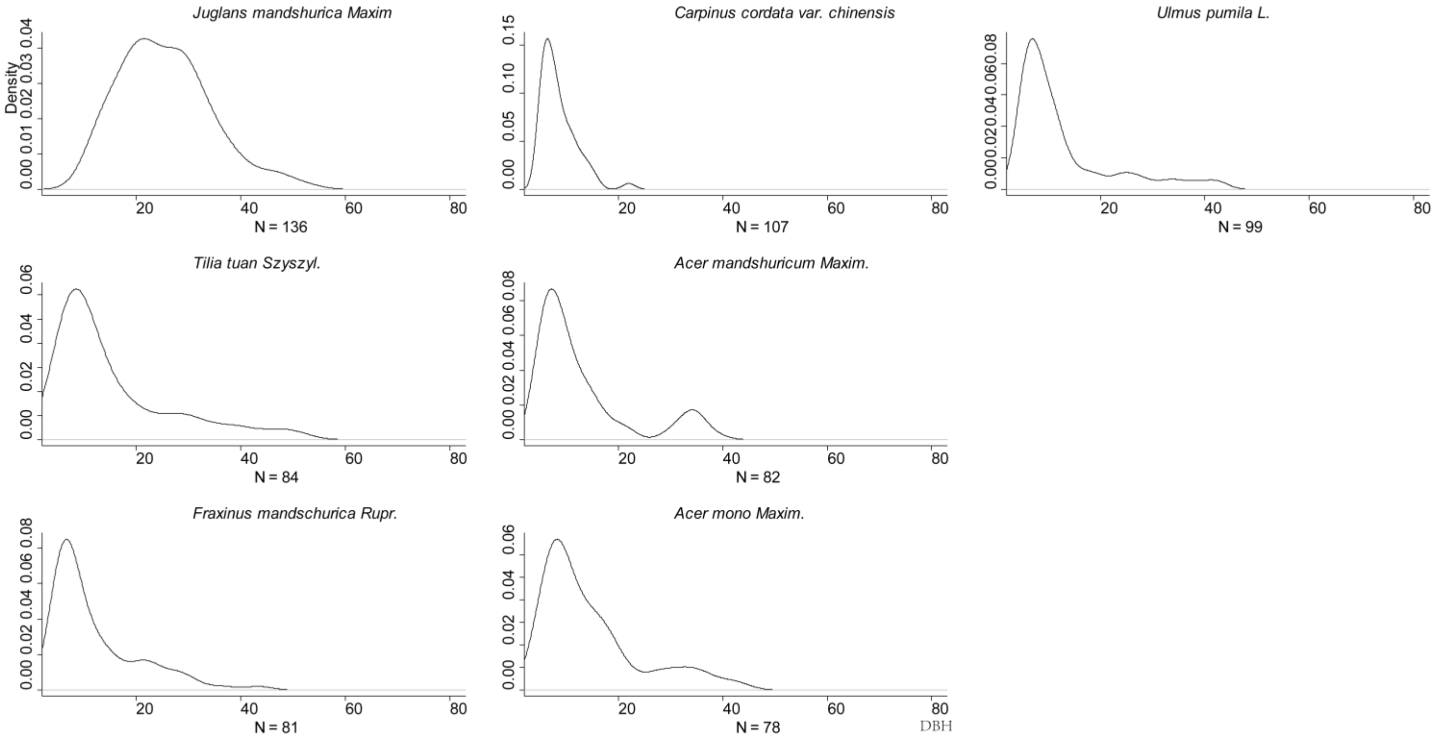

Supplementary Fig. S4 Tree dbh size distributions for species represented by more than 50 individuals in plot d.
